# Community-based Participatory Research: Necessary Next Steps

**Published:** 2007-06-15

**Authors:** Zubaida Faridi, Jo Anne Grunbaum, Barbara Sajor Gray, Adele Franks, Eduardo Simoes

**Affiliations:** Yale-Griffin Prevention Research Center, Yale School of Medicine; Prevention Research Centers Program, Centers for Disease Control and Prevention, Atlanta, Ga; Prevention Research Centers Program, Centers for Disease Control and Prevention, Atlanta, Ga; Prevention Research Centers Program, Centers for Disease Control and Prevention, Atlanta, Ga; Prevention Research Centers Program, Centers for Disease Control and Prevention, Atlanta, Ga

## Abstract

Community-based participatory research (CBPR) is gaining increasing credence among public health researchers and practitioners. However, there is no standardization in assessing the quality of research methods, the effectiveness of the interventions, and the reporting requirements in the literature. The absence of standardization precludes meaningful comparisons of CBPR studies. Several authors have proposed a broad set of competencies required for CBPR research for both individuals and organizations, but the discussion remains fragmented. The Prevention Research Centers (PRC) Program recently began a qualitative assessment of its national efforts, including an evaluation of how PRCs implement CBPR studies. Topics of interest include types of community partnerships; community capacity for research, evaluation, and training; and factors that help and hinder partner relationships. The assessment will likely contribute to the development of a standard set of competencies and resources required for effective CBPR.

## Introduction

Community-based participatory research (CBPR) has captured the interest of public health researchers and communities alike, because it promises to generate health-enhancing programs well positioned for ready adoption by communities. The seminal work of Kurt Lewin (1) and Paul Freire (2) — to name just two early researchers — dates back to the 1930s and emphasizes an iterative process of action, reflection, and experiential learning. This process is essentially the foundation of CBPR as it is practiced today. Ten years ago, the Institute of Medicine (IOM) recommended CBPR as one of eight new areas in public health education (3). Despite that recommendation, it is unclear how widespread CBPR implementation is within schools of public health. In addition, the CBPR field lacks accepted research designs and outcome measures to determine the effectiveness of the approach. Few established guidelines enumerate the core competencies for organizations and individuals to successfully conduct CBPR.

The Prevention Research Centers (PRC) Program is a large extramural research initiative at the Centers for Disease Control and Prevention. Congress authorized the PRC Program in 1984 to conduct applied public health research, and the first three PRCs were funded in 1986. Currently, 33 PRCs are located in schools of public health or schools of medicine with an accredited preventive medicine residency program. This network of academic research centers collaborates with public health agencies and community members to conduct applied research in disease prevention and control, generally in underserved communities.

In 1997, the IOM conducted a review of the PRC Program and identified areas of strength and areas needing improvement (4). One area for improvement reflected the emerging recognition that the community is an important factor in the health of individuals. The IOM review indicated that "PRCs could serve as leaders in building partnerships, if they are able to progress to a second phase that involves research and dissemination projects that are jointly planned and produced with community partners who have joint ownership of the programs" (4). While many PRCs partnered with their communities before the 1997 IOM report, it was then that the PRC Program formally integrated CBPR into its prevention research framework.

## Defining CBPR

Among the terms used to describe CBPR and its analogues are *community action research*, *participatory action research*, *community-based action research*, *participatory rapid appraisal*, and *empowerment evaluation*.

Minkler (5) described CBPR as "a process that involves community members or recipients of interventions in all phases of the research process." Green and Mercer (6) defined CBPR as "a systematic inquiry, with the collaboration of those affected by the issue being studied, for purposes of education and taking action or effecting change." Sometimes the term is applied to community-based participatory efforts to implement health enhancement programs that do not include research components at all (7).

The W.K. Kellogg Foundation Community Health Scholars Program defines CBPR as follows:

[CBPR] is a collaborative approach to research that equitably involves all partners in the research process and recognizes the unique strengths that each brings. CBPR begins with a research topic of importance to the community and has the aim of combining knowledge with action and achieving social change to improve health outcomes and eliminate health disparities (8).

The PRC Program bases its CBPR framework on that definition, and in its 2003 request for applications the PRC Program required that applicants 1) establish and maintain a center-level community committee; 2) establish and maintain partnerships with health departments, community groups and agencies, and academic units, and include these partners in center activities; and 3) collaborate with partners on planning and implementing the core research.

## Characteristics of CBPR

CBPR is an orientation to research that alters the relationship between the researchers and the research participants. In traditional research, academicians define the research issues, determine how research is done, and decide how outcomes are used. University-based departments and professional schools are generally the arbiters of who has the appropriate knowledge to define research and who is qualified to perform it. In contrast, CBPR is predicated on mutual ownership of the research process and products as well as shared decision making (9).

Translating research findings into practice is always a desired outcome, yet the rate of translation has been "inefficient and disappointing" in traditional research (10). In contrast, CBPR methodology theoretically increases the likelihood that research findings will be readily implemented in communities, because communities are invested in the preliminary testing during the research process. Because CBPR is iterative, the research process can build strong and long-lasting partnerships between researchers and research participants (11). Indeed, CBPR relies on durable partnerships that take substantial investments of time and resources to develop and sustain (11).

Efforts to summarize CBPR activity have demonstrated striking variations in methodology. The *Journal of General Internal Medicine*'s special supplement on CBPR in July 2003 included 11 original research papers that demonstrate "how broadly CBPR is being applied, geographically, within specific population groups and clinical scenarios, and methodologically" (12). For example, the settings ranged from rural to urban; the scope of research included randomized controlled trials, intervention studies with prestudy and poststudy comparisons, survey research, and qualitative methodology; and the clinical scenarios ranged from chronic disease management to cancer prevention (12).

Similarly, in a review of 185 articles of CBPR, Viswanathan et al (13) found broad variation in methods, results, and quality of research. The studies involved variable degrees of community participation, from research idea generation to project-specific advisory roles, as well as differences in other characteristics, such as outcome measures, definitions of success, and rigor of research methodology — from randomized, controlled trials to nonintervention studies. The authors noted that the nonexperimental design of most CBPR studies impedes the generalizability of findings (13).

Proposed measures of success in CBPR have included completion of a research component, increased community capacity to address the problem, successful partnership, and sustainability of the project. Vishawanathan et al recommend that CBPR projects be assessed on the degree of "co-learning" by both researchers and community collaborators (13). O'Toole et al have lamented the lack of high-quality reports for CBPR studies and suggest that a common language for reporting findings would be helpful (12).

These analyses reflect the status of CBPR and highlight gaps in the field. Deficits that have emerged include the lack of 1) common terminology, outcome measures, and an evaluation framework, which are necessary to compare CBPR studies, and 2) a structured and systematic list of essential competencies for CBPR at both the individual and organizational levels.

## Competencies for CBPR

Several authors and institutions have proposed a broad set of competencies necessary for CBPR researchers. The Kellogg Community Health Scholars program lists items such as understanding the mission and the values of CBPR; knowing theoretical frameworks, models, and methods of planning, implementation, and evaluation of CBPR; and being able to translate the process and findings of CBPR into policy (14).

Whitmore et al (15) list several questions that organizations and individual researchers should consider before embarking on a CBPR project, including whether the research team has the necessary skills to conduct the project. Also important is whether the institution has the requisite resources and infrastructure to engage in this type of research. Standardization of core competencies would allow organizations to evaluate how well their skills and resources would match with this methodology and would advance the field of CBPR.

Israel et al (16) have proposed a list of training and experience as well as personal qualities required to be a CBPR researcher — for example, ability to be self-reflective and admit mistakes, capacity to work within different power structures, and humility. Seifer et al (17) emphasize the need for interpersonal and facilitation skills, sensitivity to community needs, good communication skills, technical skills (such as grant writing and program evaluation), connections to the community, and commitment to the partnership process. Despite the guidance offered by these resources, the lists are neither comprehensive nor uniform (17).

Even less guidance is available on the institutional capabilities necessary to support and sustain CBPR. Few experts provide details on the time, energy, resources, funding mechanisms, tenure structures, organizational hierarchy, research focus, power-sharing arrangements, and institutional commitment required to conduct CBPR and maintain successful partnerships with communities. Practitioners of CBPR have addressed some of these points, but the discussion remains fragmented (18,19).

## Qualitative Assessment of the PRC Program

To provide a better understanding of partnerships, organizational factors, and the value added by CBPR, the PRC Program launched a qualitative assessment of its national efforts in the fall of 2006. One aspect of the assessment was to describe the implementation of CBPR since 2003 and answer the question, "How do PRC researchers and their communities interact to develop, implement, evaluate, and disseminate the core prevention research project?" Three key topics were explored: 1) types of community partnerships and levels of involvement, including the capacity of community committees for research, evaluation, and training; 2) types of participation in PRC research by community committee members and key partners, including factors that help and hinder partner relationships; and 3) perceived benefits of being in the PRC network as viewed by community members. Two additional questions, one related to organizational factors and one related to training, technical assistance, and mentoring, cover topics for understanding the PRCs' approaches to CBPR.

Data collection took place from January 2007 through June 2007 and included 1-hour interviews with PRC directors and principal investigators, training coordinators, and community committee chairs. For each topic area, data were collected from a carefully chosen sample of PRCs, which helped ensure that a range of CBPR approaches were covered.

The qualitative assessment will provide a wide range of descriptive information on PRC partnerships and CBPR approaches and strategies, including the number and types of community committees, the development and evolution of community partnerships, the involvement of partners and community members in core prevention research projects, and methods used to ensure that partners and community members have input into core research. The assessment will also provide models that can be used for partner and community involvement in research; university support for community-based work; and training, technical assistance, and mentoring activities. Future studies need to determine the characteristics and capacities of researchers, academic institutions, and community organizations involved in successful CBPR projects. This information will make it easier to develop a comprehensive inventory of competencies required to conduct CBPR.

## Conclusion

CBPR has been referred to as "research plus" (12) in that it not only increases the knowledge base for public health but also promises to identify interventions that are ready for dissemination and are sustainable because they have been developed with community engagement. A review of the quantity and quality of the CBPR literature reveals a picture as varied as the projects, the researchers, and the communities involved. Such extreme variation in methods and quality does not generate a useful body of knowledge. It is thus timely and imperative to delineate a core set of skills and expertise required to be a CBPR researcher and describe the essential resources and organizational infrastructure needed to successfully support CBPR. Standardizing the evaluation measures will enhance the scientific rigor of the research methods employed and improve the field's ability to study, understand, and rectify complex community health problems. The qualitative assessment of CBPR projects within the PRC Program has the potential to accelerate this process. Once an agreed-upon set of competencies and resources is established, assessment of CBPR itself can begin.
